# Fecal microbiota changes in NZB/W F1 mice after induction of lupus disease

**DOI:** 10.1038/s41598-021-02422-9

**Published:** 2021-11-25

**Authors:** Yen-Fu Chen, Ao-Ho Hsieh, Lian-Chin Wang, Yun-Ju Huang, Yun-Chen Tsai, Wen-Yi Tseng, Yu-Lun Kuo, Shue-Fen Luo, Kuang-Hui Yu, Chang-Fu Kuo

**Affiliations:** 1 Division of Rheumatology, Allergy and Immunology, Chang Gung Memorial Hospital, Taoyuan, Taiwan; 2 Division of Rheumatology, Allergy and Immunology, Chang Gung Memorial Hospital, Keelung, Taiwan; 3 Biotools Co., Ltd, New Taipei City, Taiwan

**Keywords:** Experimental models of disease, Microbiome, Systemic lupus erythematosus

## Abstract

The association between the gut microbiota and the development of lupus is unclear. We investigated alterations in the gut microbiota after induction of lupus in a murine model using viral peptide of human cytomegalovirus (HCMV). Three treatment arms for the animals were prepared: intraperitoneal injection of HCMVpp65 peptide, adjuvant alone, and PBS injection. Feces were collected before and after lupus induction biweekly for 16S rRNA sequencing. HCMVpp65 peptide immunization induced lupus-like effects, with higher levels of anti-dsDNA antibodies, creatinine, proteinuria, and glomerular damage, compared with mice treated with nothing or adjuvant only. The Simpson diversity value was higher in mice injected with HCMVpp65 peptide, but there was no difference in ACE or Chao1 among the three groups. Statistical analysis of metagenomic profiles showed a higher abundance of various families (*Saccharimonadaceae*, *Marinifiaceae*, and *Desulfovibrionaceae*) and genera (*Candidatus Saccharimonas*, *Roseburia*, *Odoribacter*, and *Desulfovibrio*) in HCMVpp65 peptide-treated mice. Significant correlations between increased abundances of related genera (*Candidatus Saccharimonas*, *Roseburia*, *Odoribacter*, and *Desulfovibrio*) and HCMVpp65 peptide immunization-induced lupus-like effects were observed. This study provides insight into the changes in the gut microbiota after lupus onset in a murine model.

## Introduction

Systemic lupus erythematosus (SLE) is a prototype of systemic autoimmune diseases characterized by persistent chronic inflammation and production of autoantibodies, particularly anti-dsDNA antibodies. It also results in dysregulation of cytokines, leading to severe and irreversible tissue injury^1,2^. Although the etiology is uncertain, genetic, environmental, hormonal, and epigenetic factors are associated with SLE development^3–5^. The mammalian gut is colonized by trillions of microorganisms that shape intestinal microbial diversity, collectively known as the microbiota^6^. The reciprocal interplay between the intestinal microbiota and the host immune system maintains tissue homeostasis^7–9^. Therefore, autoimmune diseases such as SLE may be associated with changes in the gut microbiota.

A recent cross-sectional study confirmed decreases in species richness diversity and taxonomic complexity in the feces of lupus nephritis patients compared with controls^10^. Other human evidence also links gut microbiota changes to the presence of serum antinuclear antibodies and changes in inflammatory cytokines associated with SLE progression^11,12^. In animal studies, altered microbial community structure and greater bacterial diversity have been reported in SLE^13,14^. Increased relative abundances of *Lachnospiraceae* and *Rikenellaceae* were reported to be associated with the severity of murine lupus, suggesting that the gut microbiota significantly influences the host immune system and effectively affects the development of SLE^15,16^. The interplay between dietary tryptophan intake and microbial dysbiosis in lupus-susceptible mice could contribute to the exacerbation of lupus^17^. Pattern changes in intestinal microorganisms or the presence of specific bacterial genera in the gut are associated with immune responses related to lupus.

Human cytomegalovirus (HCMV), a virus linked to the development of SLE in humans, accelerates lupus-like disease in murine models^18,19^. Anti-dsDNA antibody production, proteinuria, and glomerular attack have been reported in mice that received CMVpp65 or its fragment^18,20^. In this study, we used HCMVpp65 peptide to immunize NZB/W F1 mice to induce lupus-like effects. We investigated the dynamics of the fecal microbiota associated with lupus-like effects in HCMVpp65_422-439_-immunized mice, compared with mice treated with PBS or adjuvant only.

## Results

### HCMVpp65_422-439_ immunization induces lupus-like activity in NZB/W F1 mice

**Figure 1:**
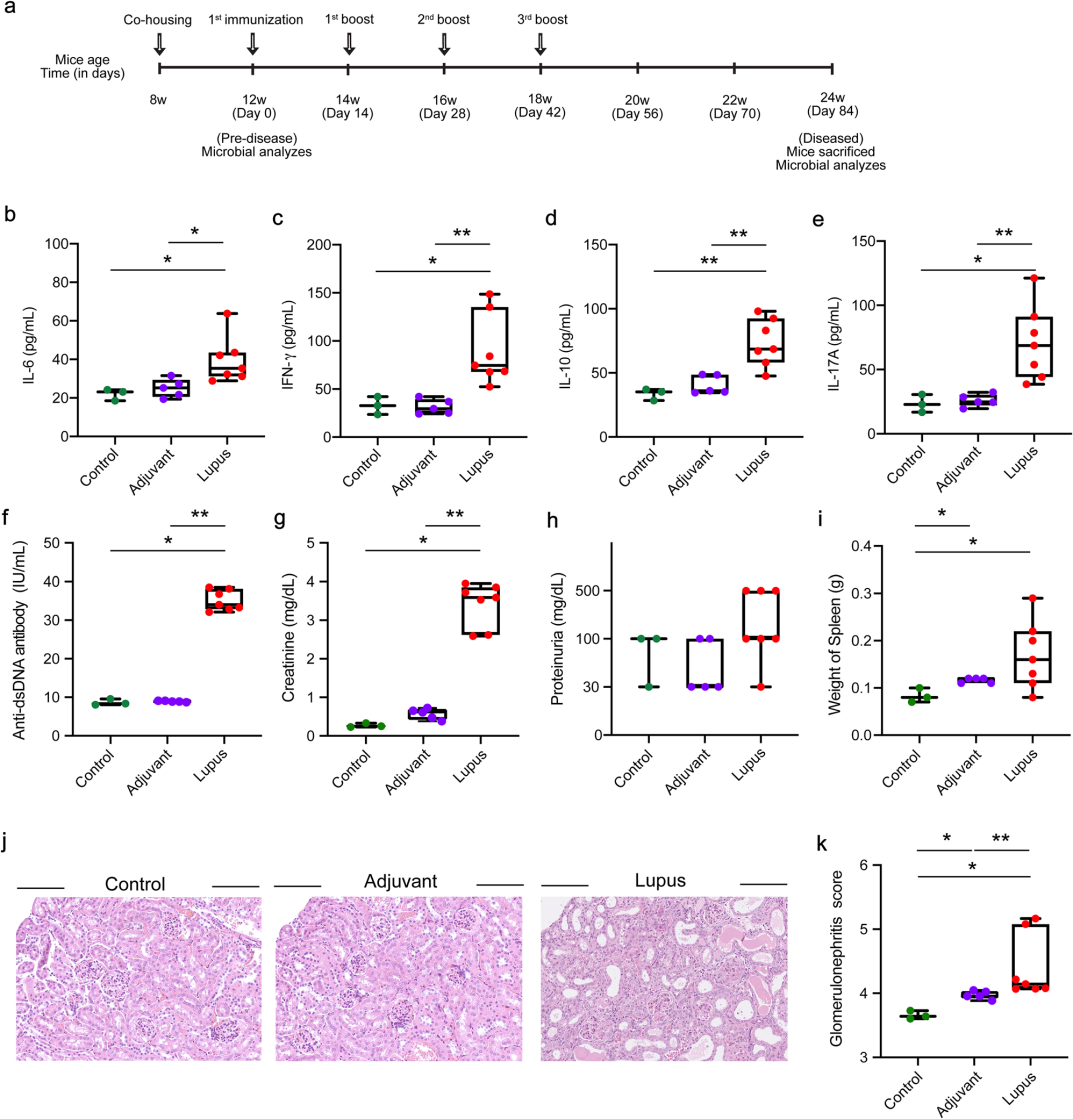
Induction of lupus-like effects in NZB/W F1 mice. Mice were intraperitoneally injected with HCMVpp65_422-439_ peptide, adjuvant, or PBS only. (**a**) Schematic of the experiment. Serum (**b**) IL-6, (**c**) IFN-γ, (**d**) IL-10, and (e) IL-17A levels in the lupus (n = 7), adjuvant (n = 5), and control (n = 3) groups at 12 weeks post immunization (24 weeks of age). The levels of (**f**) anti-dsDNA antibody (**g**) serum creatinine, and (**h**) proteinuria in blood and urine from the control, adjuvant, and lupus groups at 12 weeks post-immunization. (**i**) Representative photograph and diagram of spleen size. Data are means ± SEM. (**j**) Hematoxylin and eosin staining of glomeruli from the control, adjuvant, and lupus groups. (**k**) Glomerulonephritis score of renal lesions. Data are means ± SEM of three independent experiments. * *P* < 0.05 and ** *P* < 0.01. Full images are shown in Supplementary Fig. 1.

HCMVpp65_422-439_ immunization induces anti-dsDNA autoantibodies and initiates glomerulonephritis in non-autoimmune prone mice^21^. To investigate alterations in fecal microbiota-associated viral peptide-induced lupus-like activities, we conducted HCMVpp65_422-439_ immunization of NZB/W F1 mice at 12 weeks of age and evaluated the lupus-like effects (Fig. 1a). The experiment timeline is shown in Fig. 1a. The serum levels of IL-6, IFN-γ, IL-10, and IL-17A were higher in HCMVpp65_422-439_-immunized mice at 12 weeks post-immunization (24 weeks of age, lupus group) compared with NZB/W F1 mice treated with PBS (control group) or adjuvant only (adjuvant group, Fig. 1b–e). Anti-dsDNA antibody, serum creatinine, and proteinuria levels were elevated in the lupus group at 12 weeks post-immunization (24 weeks of age, Fig. 1f–h). Moreover, the lupus group had larger spleens, more severe renal damage, and a higher glomerulonephritis score compared with the other two groups (Fig. 1i–k). These findings suggest that HCMVpp65_422-439_ immunization induced lupus-like effects.

### Microbial composition in NZB/W F1 mice

**Figure 2:**
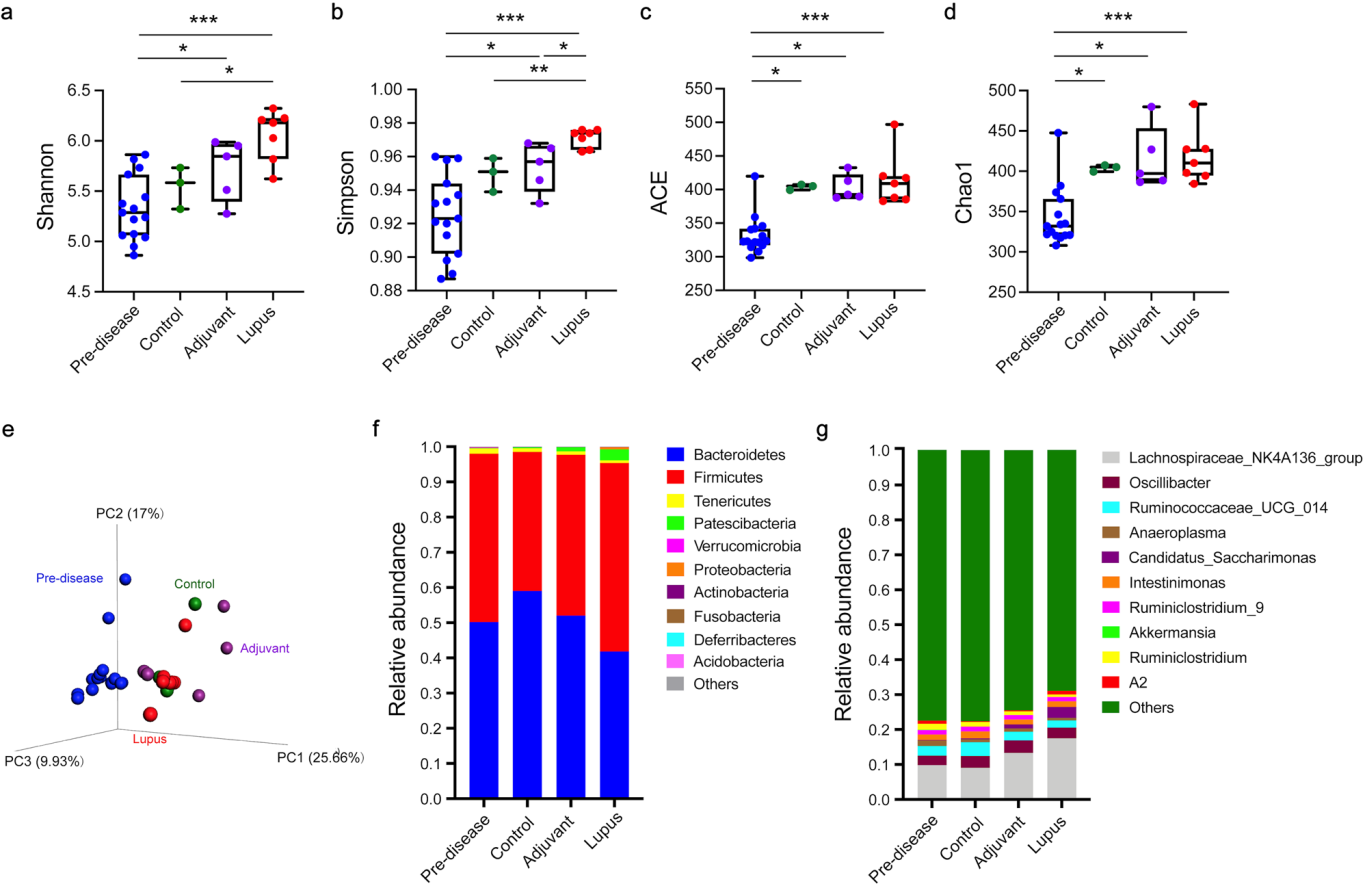
Microbial community structure. (**a**) Shannon, (**b**) Simpson, (**c**) ACE, and (**d**) Chao1 alpha diversity indices analyzed by the Kruskal–Wallis test. Statistical analysis was performed using ANOVA and Student’s *t*-test. The beta diversity of (**e**) principal coordinate analysis (PCoA) was evaluated by unweighted UniFrac and Bray–Curtis distances. Relative distributions of the top 10 (**f**) phyla and (**f**) genera. Data are means ± SEM. * *P* < 0.05, ** *P* < 0.01, and *** *P* < 0.001.

To investigate the microbial community in NZB/W F1 mice, we analyzed fecal samples collected before immunization (predisease, 12 weeks of age) and at disease time points (lupus, adjuvant, and control groups, 24 weeks of age) by 16S rRNA gene sequencing. The alpha and beta diversities were measured to evaluate alterations in microbial composition. As shown in Fig. 2a, b, the lupus group had significantly higher Shannon’s (*P* = 0.033) and Simpson’s (*P* = 0.008) diversity index values (extent of microbial diversity), but there was no difference in the ACE or Chao1 index values (extent of microbial richness), compared with the control group. A higher Simpson’s index value was found in the lupus group than in the adjuvant group. Microbial diversity and richness did not differ significantly between the adjuvant and control groups (Fig. 2a–d). For beta diversity analysis, the pattern variations in the microbial community structure among the lupus, adjuvant, and control groups are listed in Table 1. The three-dimensional principal coordinate analysis (PCoA) plot showed distinctions in community composition among the four groups (Fig. 2e). PC1, PC2, and PC3 showed 9.93%, 17%, and 25.66% of the total variance in microbial species, respectively (Fig. e). PCoA of the Bray–Curtis distance matrix revealed differences in microbial community composition among the lupus, adjuvant, and control groups (Supplementary Fig. 2). Figure 2f, g shows the relative abundances of the top 10 bacterial phyla and genera. At the phylum level, *Bacteroidetes* (59.0%) was the most abundant in the control group, followed by *Firmicutes* (39.5%), *Tenericutes* (1.0%), and *Patescibacteria* (0.3%) (Fig. 2f). The lupus group, compared with the adjuvant group, had increased abundances of *Firmicutes* (53.5% *vs*. 45.7%), *Patescibacteria* (3.1% *vs*. 1.7%), and *Proteobacteria* (0.6% *vs*. 0.007%) and decreased abundances of *Bacteroidetes* (41.8% *vs*. 52%) and *Tenericutes* (0.7% *vs*. 0.9%). At the genus level, the genera (top 10) in the relative abundance of *Lachnospiraceae NK4A136 group* (*f_ Lachnospiraceae*), *Oscillibacter* (*f_Oscillospiraceae*)*, Ruminococcaceae UCG 014* (*f_ Ruminococcaceae*)*, Intestinimonas, Candidatus Saccharimonas* (*f_ Saccharimonadaceae*)*, Ruminiclostridium*, *Ruminiclostridium 9* (*f_ Ruminococcaceae*), and *Anaeroplasma* (*f_Anaeroplasmataceae*) were observed in the four groups (Fig. 2g). The total percentages of the top 10 genera were 22.6%, 22.4%, 25.5%, and 31.0% in the predisease, control, adjuvant, and lupus groups, respectively.

**Table 1:** Statistical differences in microbial community structure between two groups.

Groups	MRPP		Adonis		Anosim	
	E-Δ	P	R^2^	P	R	P
Adjuvant *vs*. control	0.36	1.8E−2	0.21	6.3E−2	0.11	3.0E−1
Adjuvant *vs*. lupus	0.45	2.0E−3	0.30	9.0E−4	0.66	3.0E−3
Control *vs*. lupus	0.45	1.3E−2	0.35	1.4E-2	0.58	1.9E−2

Three different tests were performed using MRPP, Adonis, and Anosim and were based on the Bray–Curtis dissimilarity index. E-Δ, expected-delta.

### Altered microbial families and genera associated with HCMVpp65_422-439_ immunization

**Figure 3:**
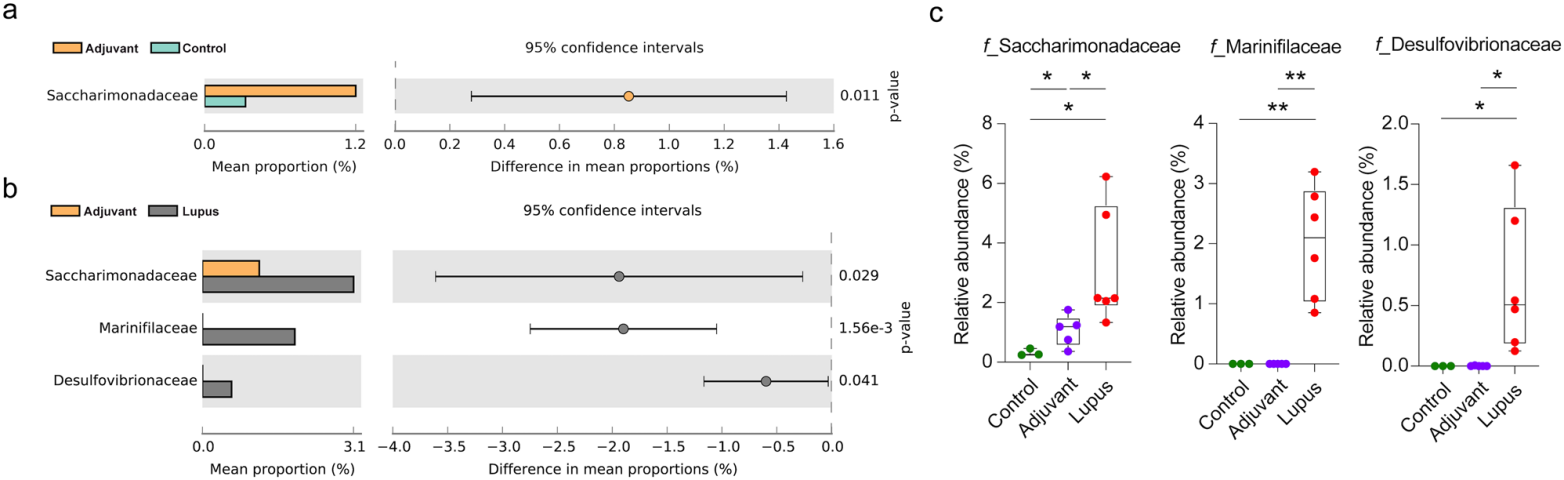
Relative abundance of fecal microbiota families. The abundances of family groups in the (**a**) adjuvant *vs*. control and (**b**) adjuvant *vs*. lupus groups analyzed by Welch’s *t*-test using STAMP software. (**c**) Families with higher abundances in the lupus compared with the adjuvant and control groups. * *P* < 0.05 and ** *P* < 0.01.

**Figure 4:**
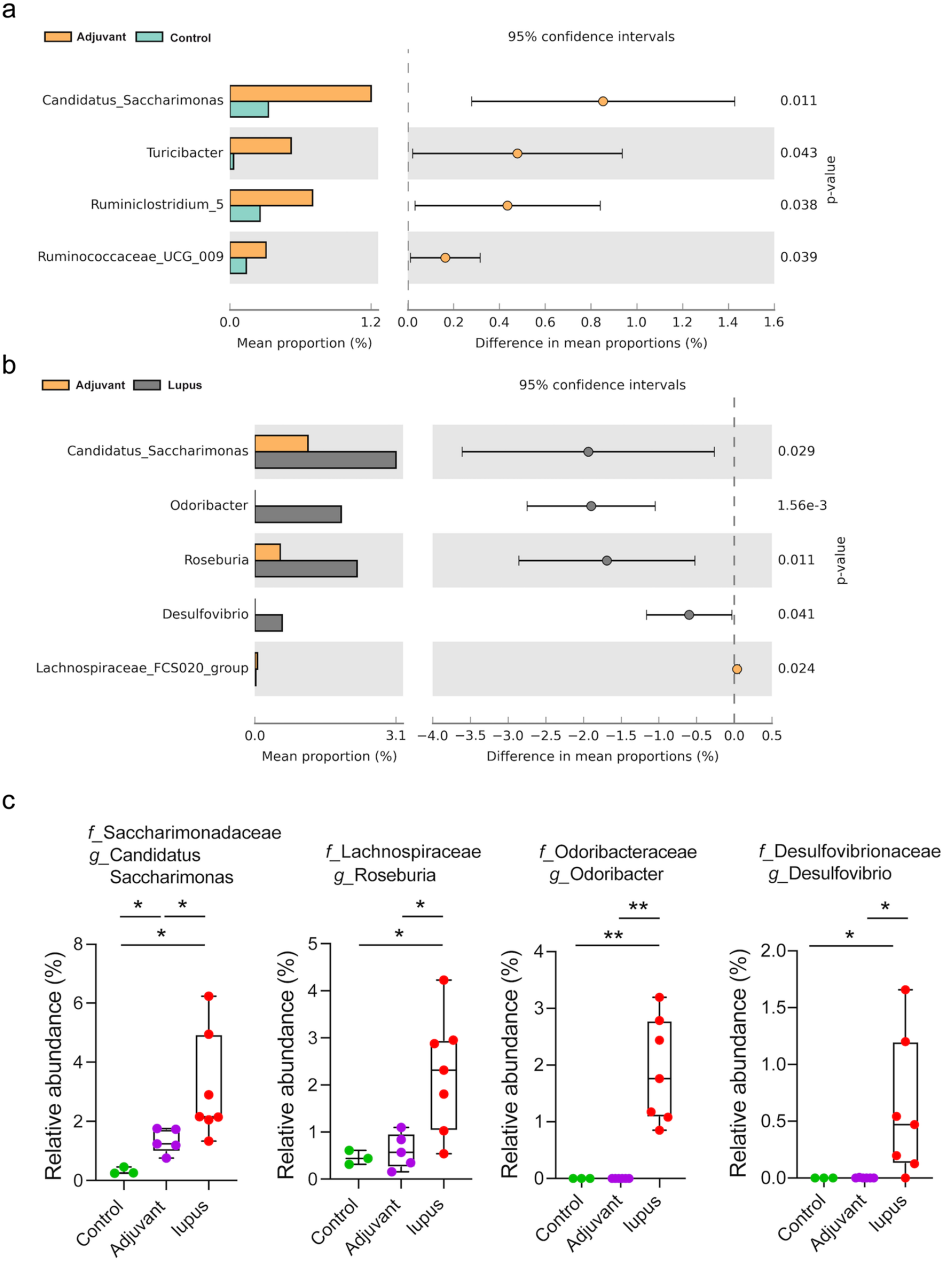
Relative abundance of genera in the fecal microbiota. The abundance of genera in the (**a**) adjuvant *vs*. control and (**b**) adjuvant *vs*. lupus groups analyzed by Welch’s *t*-test using STAMP software. (**c**) Genera with significantly higher relative abundances in the lupus compared with the adjuvant and control groups. * *P* < 0.05 and ** *P* < 0.01.

The families and genera with significant changes in relative abundance are shown in Figs. 3 and 4, respectively. At the family level, the abundance of *Saccharimonadaceae* was increased in the adjuvant group compared with control group (Fig. 3a). The lupus group had higher relative abundances of the families *Saccharimonadaceae* (3.1%), *Marinifiaceae* (2.0%), and *Desulfovibrionaceae* (0.7%) compared with the adjuvant and control groups (Fig. 3b, c). The average *Firmicutes* to *Bacteroidetes* (F/B) ratio was increased in the lupus group (1.72 ± 0.49) compared with the control (0.72 ± 0.22) and adjuvant (0.89 ± 0.23) groups (Supplementary Fig. 3a).

At the genus level, the abundances of *Candidatus Saccharimonas, Turicibacter, Ruminiclostridium 5,* and *Ruminococcaceae UCG-009* were increased in the adjuvant group compared with the control group (Fig. 4a). The abundances of *Candidatus Saccharimonas* (*f_Saccharimonadaceae*), *Odoribacter* (*f_ Odoribacteraceae*)*, Roseburia* (*f_Lachnospiraceae*), and *Desulfovibrio* (*f_Desulfovibrionaceae*) were higher in the lupus group than control group (Fig. 4b). Notably, *Candidatus Saccharimonas* (3.1%), *Roseburia* (2.3%), *Odoribacter* (1.9%), and *Desulfovibrio* (0.7%) had higher abundances in the lupus group compared with the other two groups (Fig. 4c)*.* The abundances of the families *Akkermansiaceae* and *Lactobacillaceae* were decreased in the three groups at 12 weeks post-immunization, but the difference in *Lactobacillaceae* abundance was not significant (Supplementary Fig. 3b, c). Linear discriminant analysis (LDA) effect size (> 3) showed differential abundances between the lupus *vs*. adjuvant and lupus *vs*. control group comparisons (Supplementary Fig. 4).

### Functional prediction of microbial communities associated with lupus-like effects in HCMVpp65_422-439_ immunization

**Figure 5:**
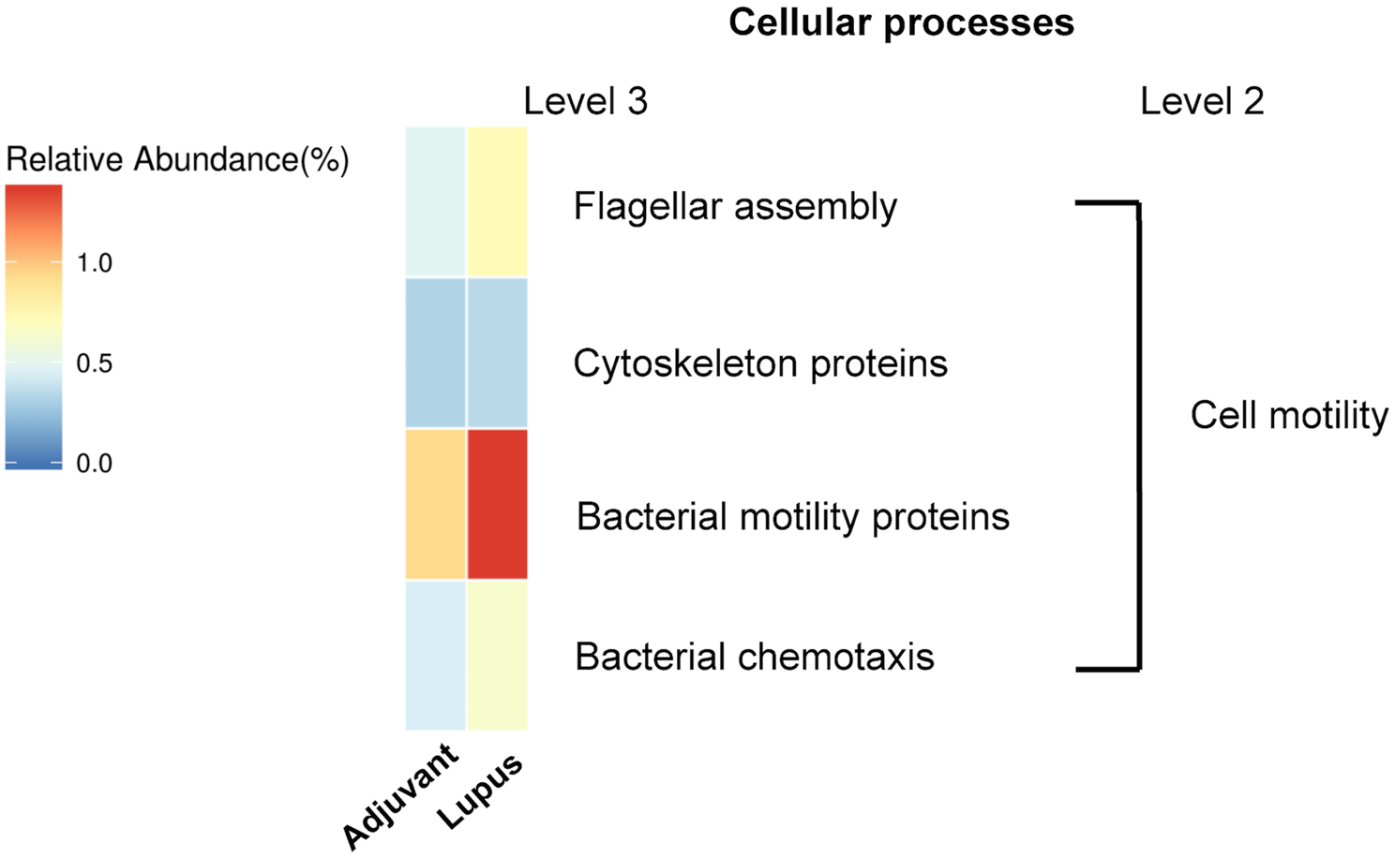
Metagenomic taxonomic profiling of microbial communities in the adjuvant and lupus groups by phylogenetic reconstruction of unobserved states (PICRUSt).

**Figure 6:**
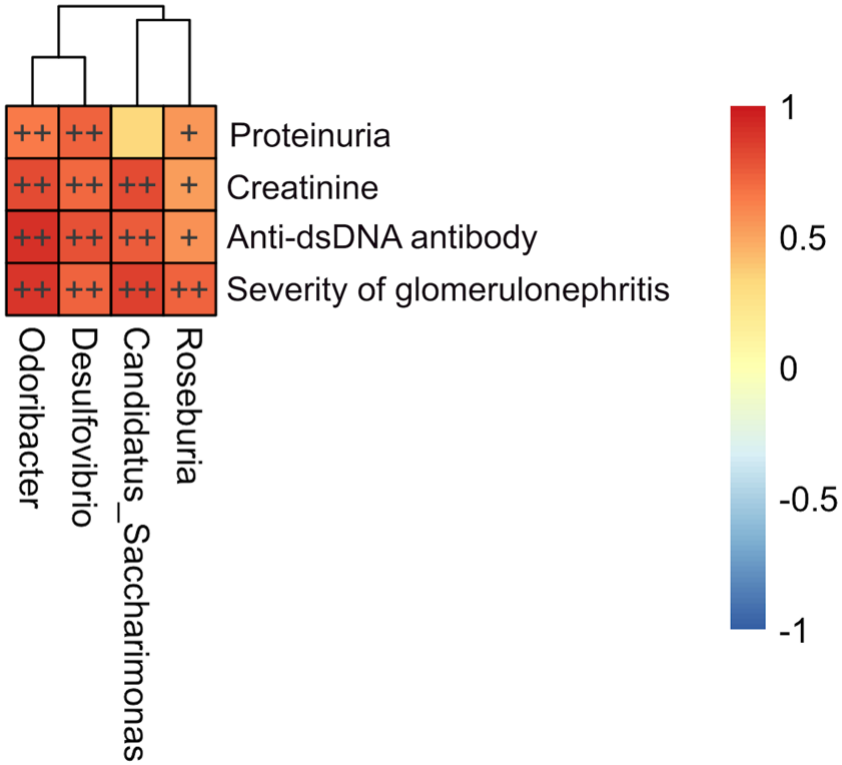
Spearman’s rank correlation between relative genera abundance and lupus-like effects in the lupus group. Heat map based on the correlation coefficient values. + *P* < 0.05 and +  + *P* < 0.01.

We predicted the functional potential of microbial communities by phylogenetic reconstruction of unobserved states (PICRUSt)^22^. Several pathways related to cell motility were differentially expressed between the adjuvant and lupus groups (Fig. 5). Also, genetic markers with significant discriminative power in cellular processes, including lysosomes, flagellar assembly, cytoskeleton proteins, bacterial motility proteins, and bacterial chemotaxis, were detected. Spearman’s rank correlation was used to infer the associations between the fecal microbial genera and lupus-like effects. Statistically significant positive correlations between microbial genera and lupus-like effects were identified (Fig. 6). All four lupus-like effects were correlated positively with *Odoribacter*, *Desulfovibrio*, and *Roseburia*. *Candidatus Saccharimonas* showed significant positive correlations with the creatinine level, anti-dsDNA IgG titer, and glomerulonephritis severity (Fig. 6).

## Discussion

Alterations in the microbiota composition are related to the development of SLE^23^. We found that fecal microbial alterations are associated with lupus-like effects in NZB/W F1 mice. HCMVpp65_422-439_ immunization accelerated lupus progression and exacerbated glomerulonephritis. The lupus group had a significantly altered microbiota composition and bacterial community compared with the adjuvant and control groups. This suggests that the abundances of significantly altered microbial families (*Saccharimonadaceae*, *Marinifilaceae*, and *Desulfovibrionaceae*) and genera (*Candidatus Saccharimonas*, *Roseburia*, *Odoribacter*, and *Desulfovibrio)* in the lupus group may be associated with HCMVpp65 peptide-induced lupus-like effects in NZB/W F1 mice.

Significant increases in the abundances of the families *Clostridiaceae*, *Lachnospiraceae*, *Ruminococcaceae*, *Desulfovibrionaceae*, and *Rikenellaceae* and a decrease in that of *Akkermansia muciniphila* have been described^24^. Moreover, the same group reported increased abundances of *Lachnospiraceae*, *Ruminococcaceae*, and *Rikenellaceae* and a decreased abundance of *Lactobacillaceae* in MRL/lpr mice at the disease stage^16^. The higher relative abundance of the *Rikenellaceae* family in SNF1 lupus mice is associated with more severe lupus symptoms^15^. Increased *Lactobacillales* in the gut of MRL/*lpr* mice was linked to improved renal function and an increased survival rate^25^. The *Lactobacillaceae* and *Akkermansiaceae* families reportedly exert an anti-inflammatory effect by increasing the IL-10 level and decreasing that of IL-6 and play a crucial role in microbiota remodeling^26–28^. In this study, the families *Saccharimonadaceae*, *Marinifilaceae*, and *Desulfovibrionaceae* were more abundant in the lupus group than the adjuvant group. The abundances of *Akkermansiaceae* and *Lactobacillaceae* were decreased in the three groups at 12 weeks post-immunization. Of note, increased abundances of *Saccharimonadaceae* and *Marinifilaceae* are positively correlated with the levels of proinflammatory cytokines such as TNF-α, IL-1β, and IL-6^29^. An association between an increased relative abundance of the *Desulfovibrionaceae* family and Th17 expression was found in patients with multiple sclerosis^30^. A significant change in phylogenetic diversity and bacterial community structure were found in the lupus group, which may be related to an increased risk of inflammatory diseases.

At the genus level, we observed increased abundances of the genera *Candidatus Saccharimonas*, *Roseburia*, *Odoribacter*, and *Desulfovibrio* in the lupus group. These microbial genera are linked to host immune regulation and autoimmune diseases. The increased abundances of *Candidatus Saccharimonas*, and *Roseburia* in colitis are reportedly associated with reduced IL-17 and TNF-α expression^31–33^. Moreover, *Roseburia* may promote regulatory T-cell differentiation and reduce the expression of IL-17 in inflammatory bowel disease^33^. In contrast, the abundances of the genera *Odoribacter* and *Desulfovibrio* are increased in several autoimmune diseases, including rheumatoid arthritis, ankylosing spondylitis, and inflammatory bowel disease^34–36^, likely due to an increased level of IL-17 and number of CD4^+^ Th17 cells. Accordingly, we examined the serum levels of IL-6, IFN-γ, IL-10, and IL-17A at 12 weeks post-immunization (24 weeks of age, disease stage). The IL-6 and IL-17A levels were significantly higher in the lupus group compared with the adjuvant and control groups. This result suggests a correlation between elevated concentrations of proinflammatory cytokines and changes in the gut microbial composition of lupus mice.

*Odoribacter* encompasses several intestinal butyrate-producing species^37^. Butyrate produced by microbial fermentation provides energy for epithelial cells, maintains the integrity of the intestinal barrier, and promotes the differentiation of regulatory T cells. Several species of the genus *Odoribacter* are reportedly decreased in abundance in patients with rheumatoid arthritis and in lupus animal models^24,38^. In addition, the abundance of *Desulfovibrio* was decreased in the gut of MRL/lpr mice, and that of *Blautia* was increased^39^. A recent study involved both an animal model and patients with anti-phospholipid syndrome (APS); in the human component, cross-reactive T/B lymphocytes and antibodies against the homogenous region expressed by the gut commensal *Roseburia intestinalis*^40^. In the animal component, immunization of normal mice with *R. intestinalis* and fecal microbiota transplantation in a murine model of spontaneous APS (NZW × BXSB) F1 induced an inflammatory reaction and cross-reactivity to beta-2-glycoprotein I. *R. intestinalis* is a trigger of APS. The discrepant results are likely due to differences in study design, enrolment criteria, and animal species and care.

The F/B ratio showed an increasing trend in NZB/W F1 mice at the disease stage (12 weeks post immunization). Firmicutes and Bacteroidetes are the predominant phyla in the human gut and have critical roles in modulating inflammation and immune status^41,42^. Bacteroidetes mediate intestinal mucosal barrier function and reduce inflammation^43,44^. Firmicutes can increase production of lipopolysaccharide, which enters the bloodstream and triggers chronic inflammation^45,46^. An elevated F/B ratio in abundant fecal microbiota is suggested to promote a proinflammatory environment and characteristics of multiple sclerosis^30,47^. The gut microbiota F/B ratio is not significantly different between SLE patients and non-SLE controls^24^. By contrast, SLE patients in remission had significantly lower F/B ratios than healthy individuals^48^. The correlation between the gut microbiota F/B ratio and disease is unclear.

This study has several limitations. Long term co-housing of mice may result in hybrid microbiota animals or cage effects. Taking advantage of coprophagy, co-housing gnotobiotic animals shortly before immunization can reduce bias in gut microbiota analysis^49^. We were unable to eliminate the cage effect, despite co-housing of mice before lupus induction. Moreover, the small population and disease severity may have caused bias. However, our results are consistent with prior reports of the microbiota composition in murine lupus models, and we observed significantly higher species and microbial community diversity. Therefore, specific bacterial community compositions may be linked to the development of autoimmune diseases.

NZBW F1 mice that received HCMVpp65 peptide developed a high anti-dsDNA antibody level, serum creatinine level, and proteinuria, which were accompanied by changes in bacterial populations at 24 weeks of age. Luo reported that the bacterial genera *Clostridium*, *Dehalobacterium, Lactobacillus, Oscillospira, Dorea* (*f_Lachnospiraceae*), *Bilophila* (*f_Desulfovibrionaceae*), and AF12 (*f*_*Rikenellaceae*) were present from the pre-disease stage (10–18 weeks of age) to the disease stage (23–33 weeks of age)^24^. The abundances of the families *Lachnospiraceae and Desulfovibrionaceae* were increased. Therefore, immunization of NZBW F1 mice with HCMVpp65 peptide not only accelerated lupus disease but also influenced the microbiota composition. Our results do not provide insight into the causality of the relationship between lupus acceleration and microbial changes, and so further studies are needed.

We investigated the association between lupus-like effects and changes in the fecal microbiota in NZB/W F1 mice. The lupus group exhibited higher microbial diversity and increased abundances of several families (*Desulfovibrionaceae*, *Saccharimonadaceae*, and *Marinifilaceae*) and genera (*Candidatus Saccharimonas*, *Roseburia*, *Odoribacter*, and *Desulfovibrio)*. The fecal microbiota composition of viral peptide-induced NZB/W F1 lupus mice differed markedly from the controls.

## Methods

### Synthetic peptides

The purity of synthetic HCMVpp65_422-439_ peptides _(GGGAMAGASTSAGRKRKS)_ was > 99%, as per the manufacturer’s guarantee (GenScript Biotech Corp, Piscataway, NJ). The HCMVpp65 peptide was prepared and stored according to the manufacturer’s recommendations.

### NZB/W F1 mice and induction of lupus-like effects

The animal experiments were approved by the Institutional Review Board of the Chang Gung Medical Foundation (#2016062804 and #2018121402). NZB/W F1 female mice (3–5 weeks old) were purchased from Jackson Laboratory Co., Ltd. and housed under specific pathogen-free conditions in the animal center at Chang Gung Memorial Hospital. After 30 days of adaptive feeding and co-housing, the NZB/W F1 mice were separated into the control group (n = 3), adjuvant group (injected with adjuvant only, n = 5), and lupus group (induced by HCMVpp65_422-439_, n = 7). The mice were housed in a specific pathogen-free room under a 12 h light/12 h dark cycle with stable humidity. The immunization schedule was as described previously^20^. Immunization was performed from 12 to 14 weeks of age. On day 1, the NZB/W F1 mice received intraperitoneal injection of 100 μg HCMVpp65_422-439_ emulsified with complete Freund’s adjuvant (Sigma-Aldrich, Catalog Number F5881). Boosting was performed using HCMVpp65_422-439_ in incomplete Freund’s adjuvant (Sigma-Aldrich, Catalog Number: F5506) or PBS on days 14, 28, and 42. All experiments were performed in accordance with relevant guidelines and regulations, including the ARRIVE guidelines.

### Serum, urine, and stool collection

Stool, urine, and blood were harvested from mice once every 2 weeks. For blood collection, mice were bled from the retro-orbital vein sinus, and plasma was collected by centrifugation at 13,000 rpm for 10 min at 4 °C and stored at − 80 °C. Fresh stool and urine samples were collected from mice and preserved in micro-tubes, which were immediately transferred to liquid nitrogen.

### Measurement of cytokines

Serum levels of IL-6, IFN-γ, IL-10, and IL-17A were measured using ELISA kits (Abnova, Taipei City, Taiwan; KA4983, KA4813, KA3070, and KA3074) according to the manufacturer’s instructions.

### Evaluation of lupus-like effects in NZB/W F1 mice

Lupus-like activity was evaluated by serum indices and kidney pathological analysis using ELISA and hematoxylin and eosin (H&E) staining^50^. Briefly, the serum level of creatinine was measured using the ELISA Test Kit (MyBioSource, MBS751125 and MBS763433, San Diego, CA). The serum anti-dsDNA autoantibody titer was evaluated using the anti-dsDNA ELISA Kit (Inova Diagnostics, catalog number: 708510, San Diego, CA) according to the manufacturers’ instructions. The proteinuria level was examined using a proteinuria strip (Medi-Test Combi 10 VET strip, MACHEREY–NAGEL, Allentown​, PA). Glomerular abnormalities were evaluated as described previously^21^. Briefly. the number of abnormalities in 100 glomeruli within a 5-μm-thick H&E-stained paraffin section of the kidney was recorded. Glomerular abnormalities were scored as follows: normal glomeruli (score 1), pure mesangial alterations (score 2), focal segmental glomerulonephritis (score 3), diffuse glomerulonephritis (score 4), diffuse membranous glomerulonephritis (score 5), and advanced sclerosing glomerulonephritis (score 6), based on the 1982 classification of the World Health Organization^51^.

### H&E staining

H&E staining was conducted according to the Cold Spring Harbor protocols with slight modifications^50^. Frozen kidney sections were immersed in 100% ethanol for 30 s and rinsed 10 times in double-distilled H_2_O. Slides were transferred to hematoxylin for 5 min and washed five times in 0.1% HCl. After washing in tap water for 5 s, the slides were washed five times in 0.1% NH_4_OH and five times in tap water. The slides were stained with eosin for 3 min and immersed in 100% ethanol with 0.1% acetic acid, 100% ethanol I, 100% ethanol II, acetone I, acetone II, xylene I, and xylene II five times each. After dehydration, the slides were mounted, covered with a cover glass, and visualized by microscopy (Olympus IX73/DP72, cellSens Standard software).

### Microbial DNA extraction and 16S rRNA gene sequencing

Microbial DNA was extracted from fecal pellets using the DNA Stool Mini Kit (Qiagen) according to the manufacturer’s instructions. The quality and quantity of DNA extracts were determined by agarose gel electrophoresis (0.8% w/v agarose) and the NanoDrop 2000 spectrophotometer (Thermo Fisher Scientific) followed by 16S rRNA sequencing.

### Data analysis and bioinformatics

Data analysis was performed using a modified protocol of Chi-Cheng Huang^52^. Amplicon sequencing was performed using 300-bp paired-end raw reads and assembled using FLASH v. 1.2.7^53^. De-multiplexing was conducted based on barcode identification. For quality control, Q < 20 reads were discarded from the pipeline of QIIME (v. 1.9.1)^54^. If three consecutive bases had Q < 20, the read was classified as truncated. The read was preserved in the data set if it contained > 75% of the original length using split_libraries_fastq.py script in QIIME^55^. Using UCHIME algorithms, sequences were checked for chimeras to acquire the effective tags and were filtered from the data set using the UPARSE function in the peptide of USEARCH (v. 7)^56–59^. The operational taxonomic unit (OTU) abundance was normalized to the variation and rarefied to the minimum sequence depth using the QIIME script (single_rarefaction.py). Subsequently, analysis of alpha and beta diversities was performed using the normalized data. Alpha diversity indicated the species complexity within individual samples based on seven different criteria outputs from the QIIME pipeline, including observed OTUs and the Shannon, Simpson, Chao1, and ACE indices^60^. Observed OTUs reflect the number of different species identified in the microbial community. The Chao1 and ACE indices used to evaluate community richness and the relative abundance and evenness accounting for diversity were assessed using the Shannon and Simpson indices. Beta diversity analysis was conducted to evaluate the differences in species complexity across samples. PCoA was performed using the distance matrix to acquire principal coordinates for visualization of sophisticated and multi-dimensional data^61^. The PCoA of the Bray–Curtis distance was analyzed according to OTU level for microbiota beta diversity.

For statistical analysis, the significance of all microbial species within groups at various levels of taxonomy was detected by differential abundance analysis using a zero-inflated Gaussian log-normal model executed in the “fitFeatureModel” function of the Bioconductor metagenomeSeq package^62^. Welch’s t-test was performed using Statistical Analysis of Metagenomic Profiles (STAMP) software (v. 2.1.3)^63^. Anosim and MRPP analyses were used to determine whether the community structures significantly differed among and within groups. Significant biomarkers were evaluated by LDA of effect size using the non-parametric factorial Kruskal–Wallis rank sum test and LDA to assess differences in taxon abundance between two groups. For functional analysis, functional abundances from 16S rRNA sequencing data were analyzed to predict functional genes using PICRUSt (v. 1.1.1)^22^. Two-tailed and unpaired Fisher’s tests and paired Student’s *t*-test with graphs depicting the mean ± three standard errors of the mean (SEM) were used for comparisons between two groups. All statistical tests were conducted with a two-sided 5% level of significance (* *P* ≤ 0.05; ** *P* ≤ 0.01; *** *P* ≤ 0.001) using SAS statistical software (v. 9.4, SAS Institute).

## Supplementary Information

Supplementary Information.

## Data Availability

The datasets presented in this study can be found in online repositories (Sequence Read Archive data, accession: PRJNA693398).
