# Supplementary material for: Fecal microbiota changes in NZB/W F1 mice after induction of lupus disease
Source: Sci Rep. 2021 Nov 25;11:22953. doi: 10.1038/s41598-021-02422-9 (PMC8616951; doi:10.1038/s41598-021-02422-9)
Supplement: Supplementary file 1 — Supplementary Information. [file 41598_2021_2422_MOESM1_ESM.docx]

**Fecal Microbiota Changes in NZB/W F1 mice after Induction of Lupus Disease**

Yen-Fu Chen^1*^, Ao-Ho Hsieh^1*^, Lian-Chin Wang^1^, Yun-Ju Huang^1^, Yun-Chen Tsai^1^, Wen-Yi Tseng^2^, Yu-Lun Kuo^3^, Shue-Fen Luo^1^, Kuang-Hui Yu^1**^, Chang-Fu Kuo^1**^

^1^ Division of Rheumatology, Allergy and Immunology, Chang Gung Memorial Hospital, Taoyuan, Taiwan

^2^ Division of Rheumatology, Allergy and Immunology, Chang Gung Memorial Hospital, Keelung, Taiwan

^3^ Biotools Co., Ltd, New Taipei City 221, Taiwan

Supplemental Fig. 1


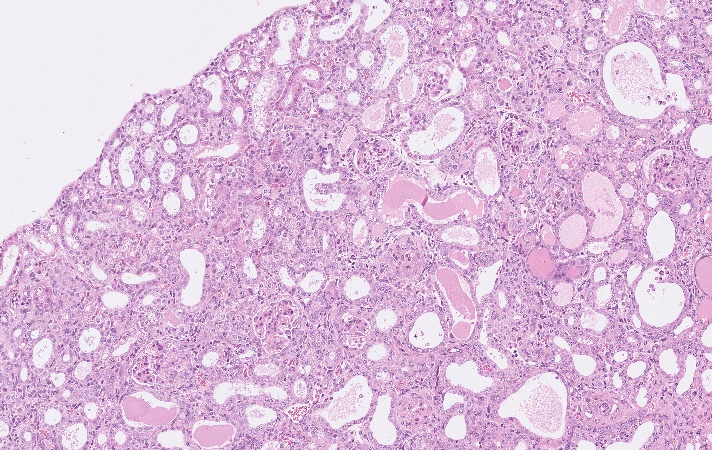


Lupus


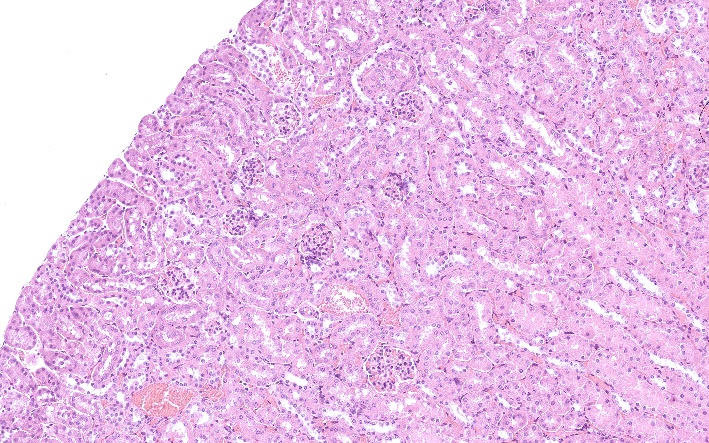


Adjuvant


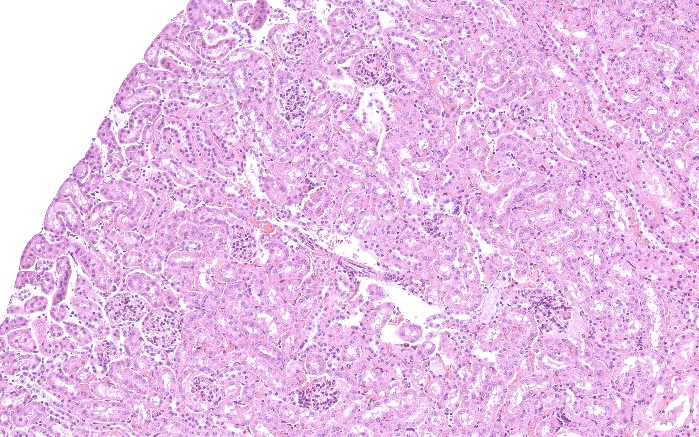


Control

**Supplementary Figure 1**. Full images of immunohistochemistry staining. Representative of full images of renal tissue sections stained with H&E from control, adjuvant, and lupus groups.


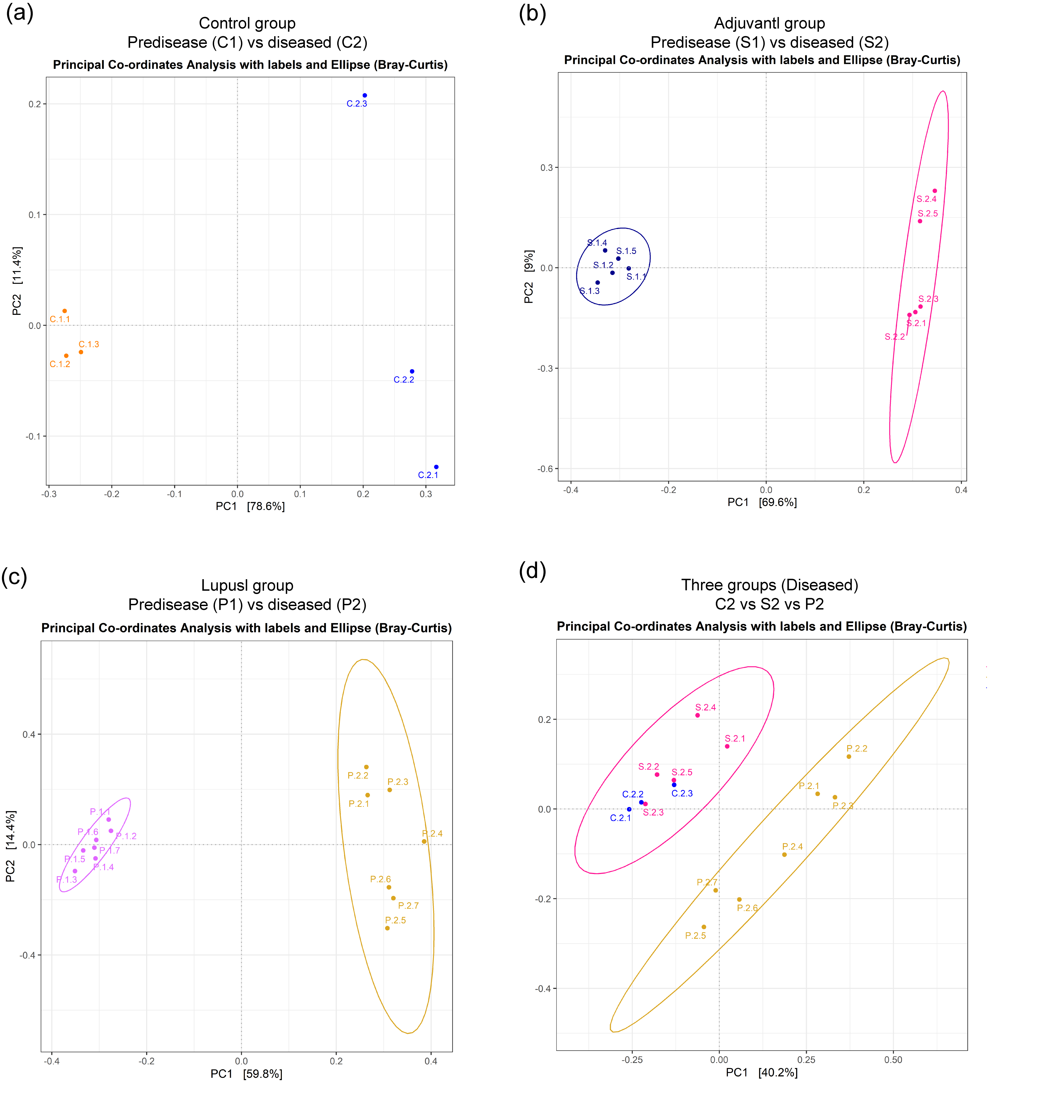
Supplementary Fig. 2

**Supplemental Fig. 2**

**PCoA of the Bray–Curtis distance matrix showing the differences in microbial community composition among the lupus, adjuvant, and control groups at the pre-disease (before immunization) and disease stage (12 weeks post-immunization).** **(a)** control (C1 vs C2, n=3), **(b)** adjuvant (S1 vs S2, n=5), **(c)** lupus (P1 vs P2, n=7), and **(d)** three groups at the disease stage (C2 vs S2 vs P2). C1: control group at the pre-disease stage, C2: control group at the disease stage, S1: adjuvant group at the pre-disease stage, S2: adjuvant group at the disease stage, P1: lupus group at the pre-disease stage, and P2: lupus group at the disease stage.

Supplementary Fig. 3


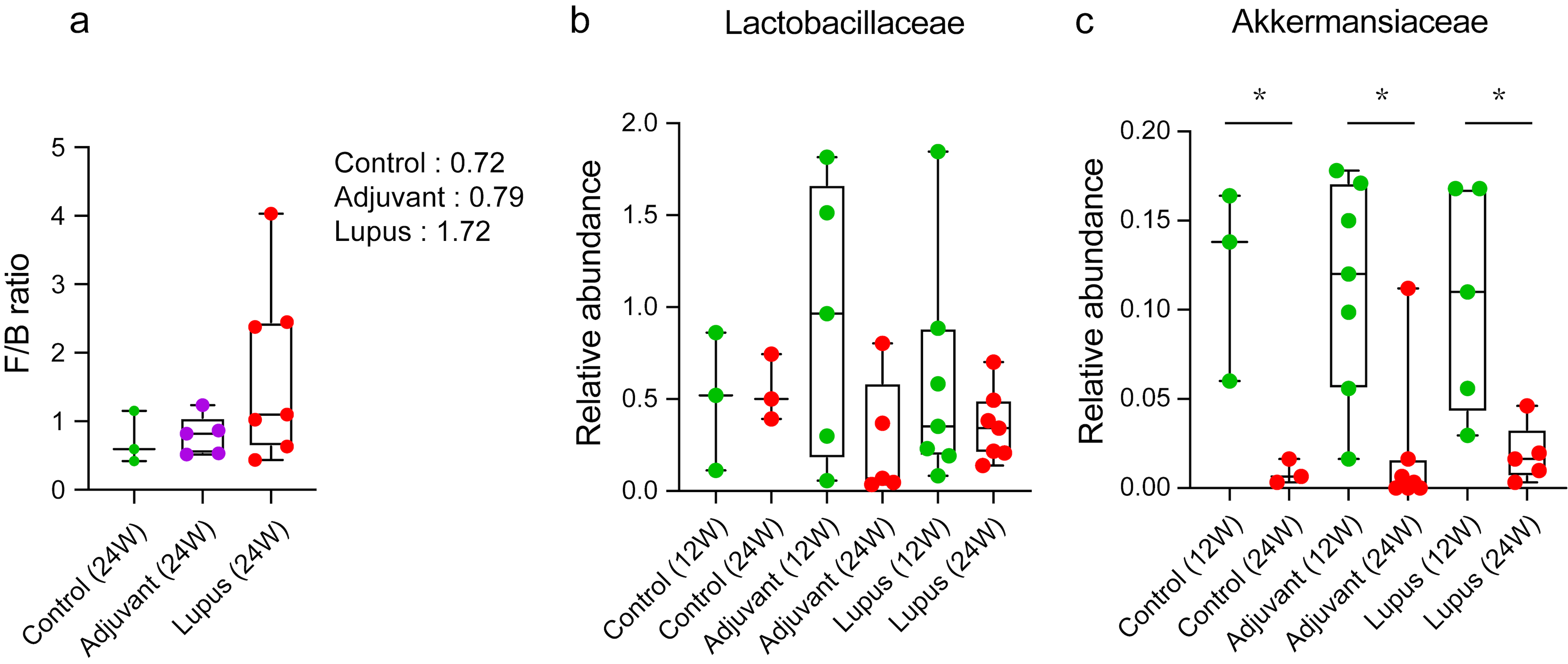


**Supplemental Fig. 3**

**Comparison of relative abundance of family *Lactobacillaceae* and *Akkermansiaceae* in the microbiota of control, adjuvant, and lupus groups. (a)** The value of F/B ratio in control, adjuvant, and lupus groups. The relative abundance of **(b)** *Lactobacillaceae* and **(c)** *Akkermansiaceae* among three groups. 12W: 12 weeks of age, pre-disease stage, 24W: 24 weeks of age, disease stage.

Supplemental Fig. 4


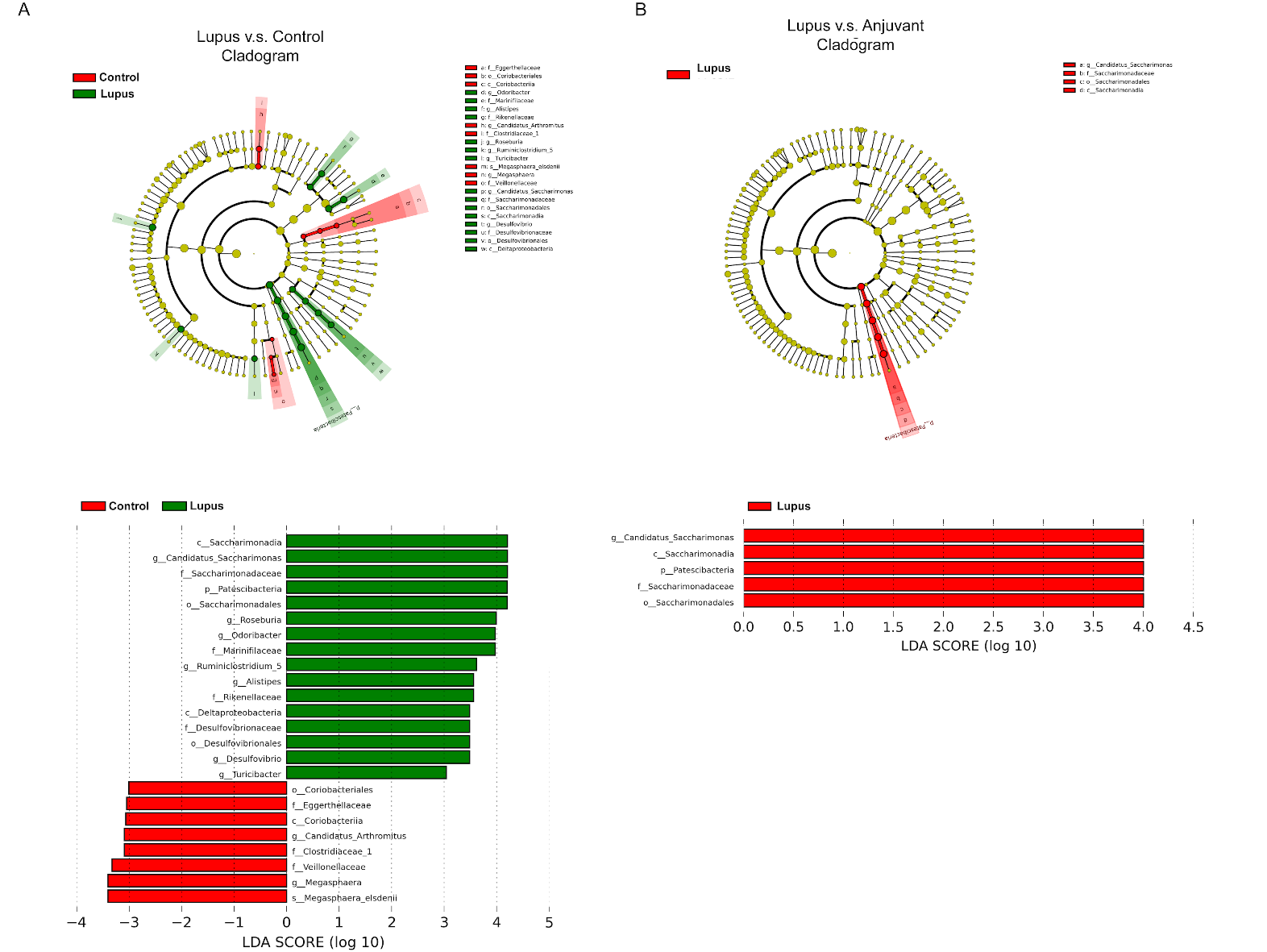


a

b

**Supplemental Fig. 4**

LEfSe was conducted to determine the features of different abundances of the **(a)** lupus vs control and **(b)** lupus vs adjuvant, and the respective effect size was evaluated by LDA. The significant differences are shown (LDA > 3)
